# Association of the Plasma Long Non-coding RNA *MEG3* With Parkinson's Disease

**DOI:** 10.3389/fneur.2020.532891

**Published:** 2020-11-26

**Authors:** Yi Quan, Jia Wang, Shuo Wang, Jizong Zhao

**Affiliations:** ^1^Department of Neurosurgery, Beijing Tiantan Hospital, Capital Medical University, Beijing, China; ^2^China National Clinical Research Center for Neurological Diseases, Beijing, China; ^3^Center of Stroke, Beijing Institute for Brain Disorders, Beijing, China; ^4^Beijing Key Laboratory of Translational Medicine for Cerebrovascular Disease, Beijing, China

**Keywords:** Parkinson's disease, long non-coding RNAs, *MEG3*, cognitive function, non-motor symptoms

## Abstract

**Objective:** To investigate the expression level of the maternally expressed gene-3 (*MEG3*) of the free long non-coding RNA (lncRNAs) in the plasma of Parkinson's disease (PD) patients and its relationship with the disease.

**Methods:** Thirty PD patients (PD group) who treated at Xuanwu Hospital of Capital University of Medical Sciences between January 2017 and December 2019 were selected as the research objects and 30 healthy subjects were enrolled in the study during the same period as the control group. Cognitive function was assessed according to the Mini-Mental State Examination (MMSE) and Montreal Cognitive Assessment (MoCA) were used to evaluate cognitive function, Non-Motor Symptoms Scale (NMSS) was used to evaluate severity of non-motor symptoms. The relative expression of lncRNAs *MEG3* in plasma was measured by PCR, and the levels of neuron-specific enolase (NSE), nerve growth factor (NGF) and brain-derived neurotrophic factor (BDNF) in plasma were measured by ELISA, and the relationship with these all indexes was analyzed.

**Results:** The NMSS score of PD group was significantly higher than that of the control group, while the MMSE and MoCA scores were significantly lower than that of the control group (*P* < 0.05); The relative expression of lncRNAs *MEG3*, NGF and BDNF levels of PD group were significantly lower than that of the control group, and NSE level was significantly higher than that of the control group (*P* < 0.05); The H&Y stage and NMSS score in PD group were negatively correlated with the relative expression of lncRNAs *MEG3*, the levels of NGF and BDNF (*P* < 0.05), and positively correlated with NSE (*P* < 0.05); The MMSE and MoCA scores in PD group were positively correlated with the relative expression of lncRNAs *MEG3*, NGF, BDNF levels (*P* < 0.05), and negatively correlated with NSE (*P* < 0.05); The relative expression of lncRNAs *MEG3* in PD group was positively correlated with NGF, BDNF levels (*P* < 0.05), and negatively correlated with NSE (*P* < 0.05).

**Conclusion:** The expression of lncRNAs *MEG3* in the plasma of PD patients was downregulated compared to that of healthy control subjects, and its expression level was closely related to the aggravation of non-motor symptoms, cognitive decline, and PD stage. These associations may reflect the synergism of the increase of NSE and decrease of NGF and BDNF levels, highlighting plasma lncRNA *MEG3* as a new candidate biomarker of PD.

## Introduction

Parkinson's disease (PD) is a progressive neurodegenerative disease and the second most common neurodegenerative disease worldwide, following Alzheimer's disease, affecting 2–3% of the global population ≥65 years of age, and the incidence rate has been increasing steadily every year (1, 2). The number of PD patients in China was estimated at about 5 million in 2019, accounting for half of the world's total number of cases, affecting quality of life for the patients and imposing an economic burden on society (3). Clinical manifestations include resting tremor, bradykinesia, muscle rigidity, balance disorders, and postural instability. With non-motor symptoms, other symptoms appear such as REM sleep behavior disorders (or RBD), olfactory dysfunction, constipation, pain, fatigue, sleep disorders, autonomic dysfunction, and cognitive dysfunction that seriously affect the quality of life of patients; in particular, the incidence of cognitive dysfunction reaches 80% or more among patients with PD (4–6).

The main pathogenic mechanisms include phasic stimulation of dopamine receptors, non-physiological levodopa-to-dopamine conversion in serotonergic neurons, hyperactivity of corticostriatal glutamatergic transmission, and overstimulation of nicotinic acetylcholine receptors on dopamine-releasing axons (7). However, the pathogenesis of PD is complex, involving genetic, environmental, and other factors, remaining a topic of continuous research and exploration. Current research suggests that the pathogenesis of PD includes oxidative stress leading to mitochondrial dysfunction, endoplasmic reticulum stress leading to abnormal protein folding, neuroinflammation, and alterations in the microecology-gut-brain axis and genes. The central link of these mechanisms involves a multi-molecular pathway network, and synergistic effects induce the degeneration of dopaminergic neurons (8). The majority of PD patients have typical motor symptoms, and about half of the dopaminergic neurons are lost irreversibly. Therefore, mining early biomarkers is of great significance for the diagnosis, treatment, and prognosis of PD.

With the rapid development of gene detection technology, the role of epigenetic modification has emerged as an important link between genetic and environmental interactions in central nervous system diseases, becoming a hot spot in clinical research in recent years. Epigenetic modifications include DNA methylation, histone modifications, and non-coding RNA (ncRNA)-mediated expression regulation. NcRNAs do not encode proteins and are now increasingly recognized to play an important role in gene transcription and in disease. Indeed, in the last decade, unprecedented numbers of ncRNAs with novel functions have been discovered. Among the various types of ncRNAs, long non-coding RNAs (lncRNAs) play important regulatory roles in physiological processes such as neuronal differentiation, and brain development and function, along with pathological processes such as cerebral ischemia-reperfusion injury, glioma, neurodegeneration, and sex-related diseases (3, 9).

LncRNAs are protein-free transcripts, but occupy a large part of the transcription output. They are involved in the regulation of epigenetic, transcription, and post-transcriptional processing in cell homeostasis, and have attracted increased research attention in biomedicine, with particular focus of the roles of lncRNAs in normal neurodevelopment and neurogenerative diseases (including Alzheimer's disease, Huntington's disease, and PD) (10, 11). However, this field is still in its infancy, and the function of most identified lncRNAs remains unclear.

The lncRNA maternally expressed gene-3 (*MEG3*) is a maternal-encoded allele that is imprinted in a gene cluster in the distal part of murine chromosome 12, corresponding to human chromosome 14. Previous studies have shown that *MEG3* acts as a tumor suppressor; its expression is lost in a variety of cancer tissues, and overexpression of *MEG3* could inhibit tumor formation (12–14). Recent studies have shown that *MEG3* is overexpressed in patients with ischemic stroke and induced the apoptosis of neurons, but is downregulated in patients with glioma and Huntington's disease (15, 16). These findings suggest that *MEG3* may be an important epigenetic regulatory factor of the brain and neurons. To explore the potential role of the lncRNA *MEG3* in the pathogenesis of PD, in this study, we compared *MEG3* plasma expression levels in patients with PD and healthy subjects, and evaluated the relationship with clinical characteristics and disease severity.

## Materials and Methods

### Subjects

Plasma samples were obtained from 30 patients diagnosed with PD who were treated at Xuanwu Hospital of Capital University of Medical Sciences, Beijing, China (kind gift from Professor Shun Yu) between January 2017 and December 2019. All patients met the PD diagnostic criteria according to the diagnostic reference standard of the neurology branch based on United Kingdom Parkinson's Disease Society Brain Bank clinical diagnostic criteria (UKPDSBB) (17). All patients had been newly diagnosed with PD and had not received any relevant treatment before enrollment in the study. The exclusion criteria were patients with severe heart, liver, kidney, and other organ dysfunction; patients with mental illness, malignant tumor, or other central nervous system diseases; and a history of substance or alcohol abuse. In addition, 30 healthy subjects were enrolled in the study during the same period as the control group. There was no statistically significant difference in age and gender between the two groups (*P* > 0.05), and the general data were comparable (Table 1).

**Table 1:** Comparison of general information between the two sets of data.

**Groups**	**Samples**	**Age**	**Gender**		**Basic diseases**			
			**Male**	**Female**	**Arterial hypertension**	**Type 2 diabetes**		
PD group	30	67.19 ± 8.12	17	13	19	13		
Control group	30	68.63 ± 7.17	15	15	18	15		
*T/X^2^*		0.728	0.268		0.071	0.268		
*P*		0.469	0.605		0.791	0.605		
**Duration of disease (years)**	**H&Y classification**					**Motor symptoms**		
	1	1.5	2	3	4	Tremor	Non-tremor	Postural instability
4.13 ± 1.50	4	4	8	9	5	8	12	10
-	-	-	-	-	-	-	-	-
-	-	-	-	-	-	-	-	-
-	-	-	-	-	-	-	-	-

The severity of the disease in PD patients is evaluated by the modified H&Y scale. Stage 0 means no symptoms or signs. Stage 1 means unilateral limb involvement. Stage 1.5 means unilateral limb involvement with symptoms of limb muscle involvement. Stage 2 means bilateral limbs are involved but there is no balance disorder. Stage 2.5 is mildly involved both limbs with mild balance disorder. Stage 3 is moderately involved both limbs with obvious postural disorder, but can take care of themselves and turn around slowly. Stage 4 is severely affected both limbs, barely able to walk or stand independently. Stage 5 is bedridden or living in a wheelchair.

Fasting peripheral blood was collected from all subjects on the morning after admission, and plasma was separated for analysis of lncRNA *MEG3* levels. All subjects provided informed consent for participation in the study, which was approved by the hospital ethics committee.

### Polymerase Chain Reaction (PCR)

Total RNA in the plasma was extracted by the Trizol method, which was used as a template for reverse transcription to obtain cDNA. cDNA was then used as a template for real-time fluorescence PCR with the lncRNA *MEG3* upstream primer 5′-GCATTAAGCCCTGACCTTTG-3′ and downstream primer 5′- TCCAGTTTGCTAGCAGGTGA-3′, synthesized by Sangon Biotech (Shanghai) Co., Ltd. *GAPDH* served as the internal reference. The relative expression levels were calculated according to the cycle threshold (Ct) value using the formula 2^−ΔΔCt^.

### Enzyme-Linked Immunosorbent Assay (ELISA)

Plasma levels of nerve-related factors, including neuron-specific enolase (NSE), nerve growth factor (NGF), and brain-derived neurotrophic factor (BDNF), were measured by ELISA. ELISA kits were purchased from Shanghai Thermo Scientific and Biological Co., Ltd. The specific operation steps were carried out in accordance with the instructions. Within 30 min, the absorbance value at 450 nm was measured with a microplate reader (Thermo Scientific, FC type), and a standard curve was drawn based on the standard substance. Measure the corresponding sample concentration, repeat the measurement for each sample three times and take the average value as the final concentration.

### Disease Severity Evaluation

Cognitive function was assessed according to the Mini-Mental State Examination (MMSE) and Montreal Cognitive Assessment (MoCA). The Non-Motor Symptoms Scale (NMSS) was used to evaluate the severity of non-motor symptoms. The NMSS score mainly reflects the severity of the patient's non-motor symptoms, such as sleep disorders, autonomic dysfunction, cognitive and psychiatric symptoms, etc. The higher the score, the more severe the above symptoms; both MMSE and MoCA are widely used in clinical practice. The cognitive function screening scale reflects the mental state and the degree of cognitive impairment. The higher the score, the better the cognitive function, and the MMSE <27 or MoCA <17 points indicate the presence of cognitive impairment; all three scores can be used evaluation of neurodegenerative state in PD patients.

### Statistical Analysis

Measurement data are presented as the mean ± standard deviation, and were compared between the groups using Student *t*-tests. Count data are presented as percentage and were compared using the chi-square test. The plasma biochemical indices were analyzed by Spearman and Pearson correlation coefficients and multivariate Logistic regression was used to analyze the relationship between H&Y scale and other indicators. The relationship between plasma lncRNA *MEG3* levels and various quantitative indices was assessed by a linear regression model. SPSS 16.0 software was used for all statistical analyses; *P* < 0.05 was considered statistically significant.

## Results

### Comparison of Scores and Plasma Biochemical Markers Between Groups

The PD patient group included 17 males and 13 females, ranging in age from 57 to 78 years with a mean age of 67.19 ± 8.12 years. Among them, there were 19 patients with hypertension and 13 patients with diabetes. The control group comprised 15 males and 15 females, aged 55–79 years with a mean age of 68.63 ± 7.17 years, including 18 patients with arterial hypertension and 15 patients with Type 2 diabetes. There were no significant differences in age, gender, and basic diseases between the two groups (all *P* > 0.05), and the general data were comparable.

The NMSS scores of the PD group were significantly higher than those of the control group, whereas the MMSE and MoCA scores were significantly lower in the patient group. The relative expression levels of plasma lncRNA *MEG3*, NGF, and BDNF in the PD group were significantly lower than those in the control group, whereas the NSE level was significantly higher than that of the control group (Table 2).

**Table 2:** Comparison between various scores and plasma biochemical indexes between two groups.

**Variable**	**Groups**		***t***	***p***
	**PD group**	**Control group**		
NMSS score	58.38 ± 33.89	24.69 ± 17.27	4.851	0.000
MMSE score	24.63 ± 4.41	28.69 ± 0.70	4.980	0.000
MOCA score	18.44 ± 4.62	26.44 ± 1.46	9.044	0.000
*MEG3* expression level	0.57 ± 0.19	0.94 ± 0.36	4.979	0.000
NSE (μg/mL)	17.55 ± 7.13	11.08 ± 4.53	4.195	0.000
NGF (pg/mL)	25.92 ± 3.26	30.78 ± 3.54	5.531	0.000
BDNF (pg/mL)	27.38 ± 3.52	32.19 ± 3.78	5.101	0.000

### Relationship Between Disease and Plasma Markers in PD Patients

There was a significant positive correlation between the H&Y stage and NMSS score in PD patients, and a significant negative correlation between MMSE and MoCA scores. The H&Y stage and NMSS scores of PD patients were negatively correlated with the relative expression level of lncRNA *MEG3*, and with the levels of NGF and BDNF, and were positively correlated with plasma NSE levels. By contrast, MMSE and MoCA scores in PD patients were positively correlated with the levels of lncRNA *MEG3*, NGF, and BDNF, and were negatively correlated with NSE levels. In addition, the relative expression level of plasma lncRNA *MEG3* was positively correlated with NGF and BDNF levels, and negatively correlated with NSE levels (Table 3).

**Table 3:** The relationship between scores and plasma biochemical indexes in PD patients.

		**NMSS score**	**MMSE score**	**MOCA score**	***MEG3* expression level**	**NSE**	**NGF**	**BDNF**
H&Y Grade	*r*	0.594^**^	−0.824^**^	−0.592^**^	−0.559^**^	0.834^**^	−0.651^**^	−0.396^*^
	*P*	0.001	0.000	0.001	0.001	0.000	0.000	0.030
NMSS score	*r*	1.000	−0.810^**^	−0.708^**^	−0.532^**^	0.362^*^	−0.643^**^	−0.662^**^
	*P*		0.000	0.000	0.002	0.049	0.000	0.000
MMSE score	*r*	−0.810^**^	1.000	0.698^**^	0.505^**^	−0.648^**^	0.630^**^	0.597^**^
	*P*	0.000		0.000	0.004	0.000	0.000	0.001
MOCA score	*r*	−0.708^**^	0.698^**^	1.000	0.431^*^	−0.450^*^	0.449^*^	0.508^**^
	*P*	0.000	0.000		0.017	0.013	0.013	0.004
MEG3 expression level	*r*	−0.532^**^	0.505^**^	0.431^*^	1.000	−0.426^*^	0.362^*^	0.593^**^
	*P*	0.002	0.004	0.017		0.019	0.049	0.001
NSE	*r*	0.362^*^	−0.648^**^	−0.450^*^	−0.426^*^	1.000	−0.557^**^	−0.469^**^
	*P*	0.049	0.000	0.013	0.019		0.001	0.009
NGF	*r*	−0.643^**^	0.630^**^	0.449^*^	0.362^*^	−0.557^**^	1.000	0.719^**^
	*P*	0.000	0.000	0.013	0.049	0.001		0.000

** *Significantly correlated at 0.01 level (bilateral)*.

* *Significantly correlated at the 0.05 level (bilateral)*.

### Multivariate Logistic Regression Analysis of H & Y Scale and Other Indexes

All the above-mentioned factors related to H&Y scale were used as independent variables to assign values. For H&Y scale, “ ≤ 2” was regarded as mild and “>2” was regarded as moderate to severe (18). Multivariate Logistic regression analysis was performed. The results showed that age, disease course, NMSS and NSE was significantly positively correlated with disease stage, while MMSE, MoCA scores and the relative expression of lncRNA *MEG3* levels were significant negatively correlated with disease stage, and the differences were statistically significant (*P* < 0.05) (Table 4).

**Table 4:** Multivariate Logistic regression analysis of H&Y classification and other indexes.

**Relevant factor**	**β**	**SE**	**Wald χ^**2**^ value**	**OR (95%CI)**	***P-*value**
Age	1.057	0.413	6.549	2.878 (1.132 – 4.558)	0.010
Duration of disease	1.289	0.382	11.385	3.629 (1.473 – 6.052)	0.001
NMSS score	0.783	0.348	5.060	2.188 (1.440 – 3.038)	0.024
MMSE score	−1.331	0.369	13.011	3.785 (1.064 – 6.057)	0.000
MOCA score	−0.751	0.341	4.850	2.119 (1.113 – 3.036)	0.028
NSE	1.289	0.382	11.385	3.629 (1.473 – 6.052)	0.001
MEG3 expression level	−0.668	0.329	4.122	1.950 (1.205 – 2.671)	0.043

### Linear Relationship Between Plasma lncRNA *MEG3* and Other Indicators

**Figure 1:**
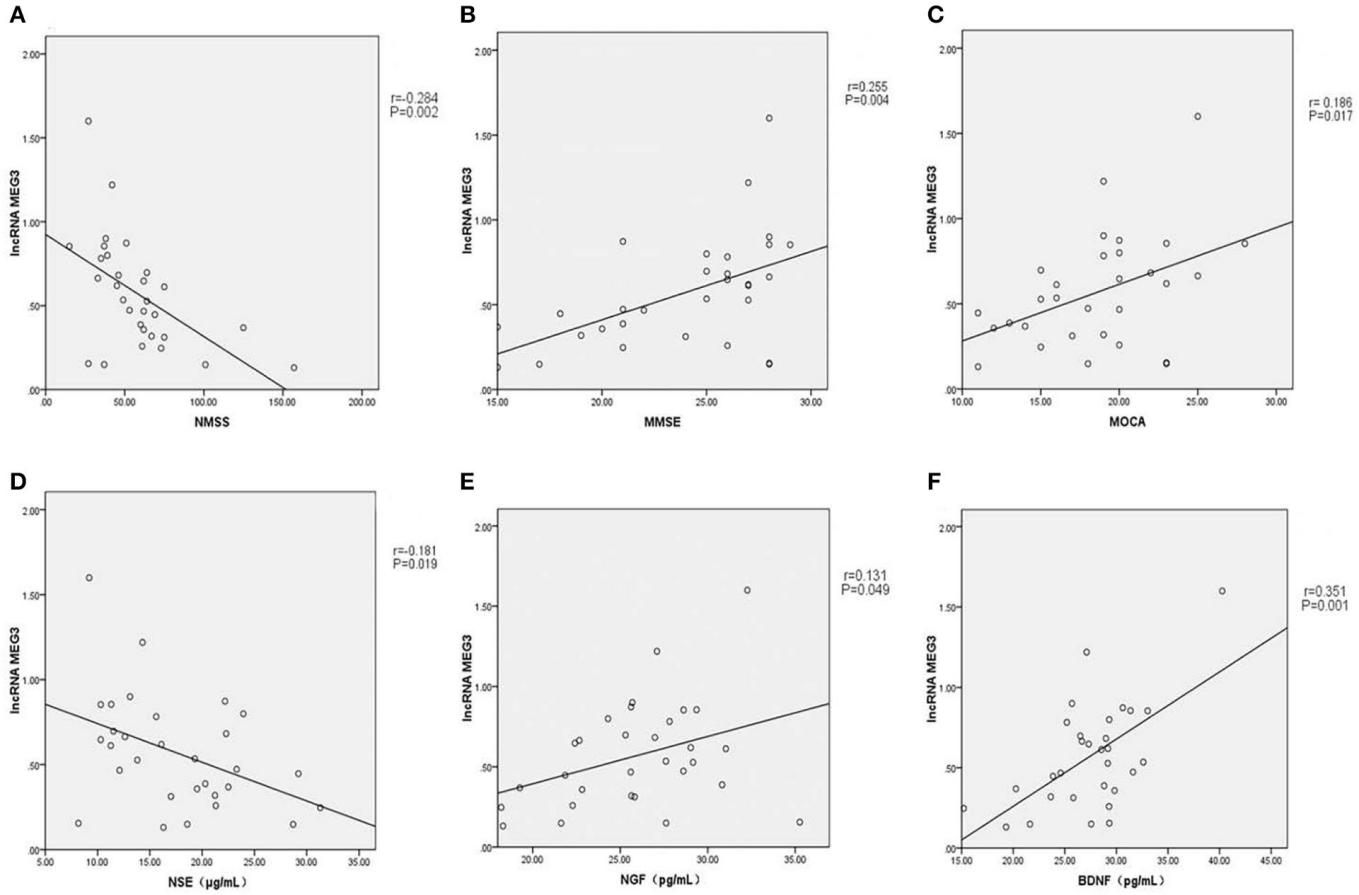
**(A)** Plasma lncRNA *MEG3* level in PD patients was negatively correlated with NMSS score (*r* = −0.284, *P* = 0.002). **(B)** Plasma lncRNA *MEG3* level in PD patients was positively correlated with MMSE score (*r* = 0.255, *P* = 0.004). **(C)** Plasma lncRNA *MEG3* level in PD patients was positively correlated with MOCA score (*r* = 0.186, *P* = 0.017). **(D)** Plasma lncRNA *MEG3* level in PD patients was negatively correlated with NSE levels (*r* = −0.181, *P* = 0.019). **(E)** Plasma lncRNA *MEG3* level in PD patients was positively correlated with NGF levels (*r* = 0.131, *P* = 0.049). **(F)** Plasma lncRNA *MEG3* level in PD patients was positively correlated with BDNF levels (*r* = 0.351, *P* = 0.001).

As shown in Figures 1A–F, linear correlation analyses showed that the plasma lncRNA *MEG3* level in PD patients was negatively correlated with NMSS score (*r* = −0.284, *P* = 0.002), and positively correlated with MMSE (*r* = 0.255, *P* = 0.004) and MoCA (*r* = 0.186, *P* = 0.017) scores. Plasma lncRNA *MEG3* levels were negatively correlated with NSE levels in PD patients (*r* = −0.181, *P* = 0.019), and positively correlated with NGF (*r* = 0.131, *P* = 0.049) and BDNF (*r* = 0.351, *P* = 0.001) levels.

## Discussion

Recent studies have shown that the lncRNA *MEG3* is associated with glioma, Huntington's disease, stroke, and other neurological disorders, suggesting a potentially new clinical biomarker (15, 16), it can block the cell cycle by activating the p53 pathway, leading to cell replication senescence or apoptosis. In this study, we compared the levels of lncRNA *MEG3* and biochemical brain markers in the plasma of PD patients with those of healthy control subjects. The results showed that the relative expression levels of plasma lncRNA *MEG3* are reduced in PD patients compared to those of the healthy population, shows that lncRNA *MEG3* is one of the most important potential molecular in the diagnosis of PD or selection of therapeutic potential targets.

The mature *MEG3* lncRNA is composed of 10 exons and is abundantly expressed in the brain, adrenal glands, placenta, breast, and liver tissues. Previous studies have demonstrated that the expression level of *MEG3* is reduced in cancer cells, and upregulation of *MEG3* expression could inhibit tumor growth; therefore, *MEG3* was initially considered to function mainly as a tumor suppressor (12, 13). Lin et al. (12) found that upregulation of *MEG3* expression in the HeLa cervical cancer cell line can inhibit the PI3K/AKT/Bcl-2/Bax/P21 signaling pathway, thus inhibiting the proliferation, invasion, and migration of HeLa cells and promoting their apoptosis. Han et al. (13) found that the lncRNA *MEG3* methylation level increased successively in the serum of healthy subjects, patients with low-stage cervical cancer, high-stage cervical cancer, and cervical cancer with lymph node metastasis, indicating that *MEG3* methylation might be a marker of disease progression in cervical cancer. Subsequently, lncRNA *MEG3* expression was found to be downregulated in glioma tissue cells. Zhang et al. (10) reported that lncRNA *MEG3* inhibited glioma cell growth *in vitro* by regulating the mir-96-5p/MTSS1 signaling pathway, and was involved in cell proliferation and apoptosis regulation. Earlier studies indicated that lncRNA *MEG3* also showed a downregulated trend in the brain tissues of patients with Huntington's disease, and *Meg3*-knockout affected the expression of genes in the cerebral cortex of mice, leading to increased cortical microvascular density and enhanced expression of genes related to angiogenesis (19). Collectively, these studies suggest that *MEG3* may also be an important epigenetic regulatory factor in brain development by regulating the gene expression profile to correspond to neuron activity. However, clinical studies on its regulatory pathway are scarce, and no consensus has been reached to date.

We found that the NMSS score of PD patients was significantly higher, whereas the MMSE and MoCA scores were significantly lower than those of the control group.

The main pathological characteristic of PD is dopaminergic neuron degeneration, which will lead to striatum dopaminergic denervation and loss of function of the dopaminergic nerve, resulting in secondary effects to the temporal lobe, cortex, thalamus, with reduced neurotransmitter synthesis and secretion in the hypothalamus, ultimately resulting in impaired cognitive function. Accordingly, in the H&Y scale of patients with PD, NMSS scores are positively correlated, whereas MMSE and MoCA scores are negatively correlated with disease severity, motor symptoms, and degree of cognitive damage (2).

NSE, BDNF, and NGEF are well-established biomarkers of cognitive impairment in PD patients, indicating that they may play a pathogenic role (20). In this study, plasma NGF and BDNF levels in PD patients were significantly lower, whereas NSE levels were significantly higher than those in the healthy controls. NSE is an acidic protease that is unique to neurons and neuroendocrine cells. When neurons in the brain are damaged in PD, a large amount of NSE enters the blood circulation; thus, the elevated level of NSE in plasma is related to disease severity. Brain tissue BDNF and NGF are neurotrophic factors (nutrients required for neuronal differentiation and development in the brain) that play roles in repairing damaged neurons and regulating synaptic functions, and participate in memory and learning; therefore, their deficiency is also closely related to the progression of neurodegenerative diseases and the occurrence of cognitive dysfunction (21, 22). We found that the H&Y stage and NMSS score were negatively correlated with NGF and BDNF levels, and were positively correlated with NSE levels. By contrast, MMSE and MoCA scores in PD patients were positively correlated with NGF and BDNF levels and negatively correlated with NSE levels, confirming these close associations with PD severity and cognitive impairment.

To our knowledge, this is the first clinical study to explore the expression of lncRNA *MEG3* in patients with PD. Methylation is one of the most well-studied epigenetic changes in degenerative diseases of the nervous system. Methylation is a dynamic process that regulates gene expression: normal cells tend to be hypomethylated, whereas brain tissues exhibit a higher level of methylation than other tissues, especially in genome repeat regions. DNA methylation typically occurs at cytosine-guanosine dinucleotide (CpG) sites with a GC content >55%. Methylation can bind transcriptional activators to DNA to inhibit gene expression or cause conformational changes in chromosomes leading to gene silencing. The 5′-end of lncRNA *MEG3* is rich in CpG dinucleotides, in which a large amount of DNA is methylated, and DNA methylation in the functional region of *MEG3* may lead to expression silencing (23). Since the brain tissue and peripheral blood show highly similar methylation modes, methylation levels in the peripheral blood can reflect those in the brain tissue. Tan et al. (24) suggested that the *SNCA* level in peripheral blood leukocytes and low *LRRK2* methylation levels can be used as potential biomarkers for PD. In addition, the levels of peripheral blood free small RNAs such as microRNAs can be used for the diagnosis of PD (25). Despite the accuracy of this approach, there are also some problems to overcome. For example, the transcription levels in the blood do not entirely reflect the local levels in the brain tissue, and expression levels can vary in different brain regions at the same time and in different conditions. In PD, the dopaminergic neurons are lost in the substantia nigra, and the blood–brain barrier can lead to low permeability. Therefore, further clinical samples from PD patients are needed to verify the free lncRNA *MEG3* expression levels (26).

We found that the relative expression level of plasma lncRNA *MEG3* was negatively correlated with the H&Y stage and NMSS score, but positively correlated with MMSE and MoCA scores, indicating that the downregulation of lncRNA *MEG3* expression may be one of the possible pathogenic mechanisms of PD. Moreover, the positive correlations between lncRNA *MEG3* and NGF or BDNF levels, and negative correlations with NSE levels suggest synergistic effects with nerve-related factors in the development and progression of PD. However, this study can provide only a preliminary discussion on the association of plasma lncRNA *MEG3* with PD, and the mechanism remains to be further elucidated with animal model experiments. In addition, due to the limitation of the number of samples, we did not rule out monogenic forms of PD, which also brings a little regret for the final result. We look forward to increasing the sample size in the later experimental verification stage and to evaluate whether different PD subtypes show different biomarker signals.

In summary, the expression of lncRNA *MEG3* in the plasma of PD patients was downregulated compared to that of healthy control subjects, and its expression level was closely related to the aggravation of non-motor symptoms, cognitive decline, and PD stage. These associations may reflect the synergism of the increase of NSE and decrease of NGF and BDNF levels, highlighting plasma lncRNA *MEG3* as a new candidate biomarker of PD.

## Data Availability Statement

All datasets generated for this study are included in the article/supplementary material.

## Ethics Statement

The studies involving human participants were reviewed and approved by Beijing Xuanwu hospital ethics committee. The patients/participants provided their written informed consent to participate in this study.

## Author Contributions

YQ had full access to all of the study data, takes responsibility for the integrity, and accuracy of the data analysis. YQ and JW: study concept, design, and statistical analysis. SW and JZ: study supervision. All authors: critical revision of the manuscript for important intellectual content.

## Conflict of Interest

The authors declare that the research was conducted in the absence of any commercial or financial relationships that could be construed as a potential conflict of interest.
